# The content of selected metals in muscles of the red deer (*Cervus elaphus*) from Poland

**DOI:** 10.1007/s11356-014-4007-0

**Published:** 2014-12-30

**Authors:** Michał Skibniewski, Ewa M. Skibniewska, Tadeusz Kośla

**Affiliations:** 1 Department of Morphological Sciences, Faculty of Veterinary Medicine, Warsaw University of Life Sciences-SGGW, Nowoursynowska 159, 02-776 Warsaw, Poland; 2 Department of Animal Environment Biology, Faculty of Animal Sciences, Warsaw University of Life Sciences-SGGW, Ciszewskiego 8, 02-786 Warsaw, Poland

**Keywords:** Red deer (*Cervus elaphus*), Muscles, Lead, Metals

## Abstract

The aim of this study was to evaluate the concentrations of Pb, Cu, Zn, Rb, Cs, Sr and Ba in the muscles of red deer that were hunted in two regions of Poland (south-western and north-eastern). The data obtained were evaluated with regards to benefits and potential risk to consumers’ health. Samples for the investigations were collected in 2008 and 2009 from 50 female red deer, and the metal concentrations were determined by using the inductively coupled plasma–mass spectrometric (ICP-MS) method. The mean concentrations of Pb did not differ statistically between regions and were equal to that permitted for farm animals. The results of this study support the conclusion that the meat of the analysed animals does not pose a risk of lead intoxication. Statistically higher mean concentrations of Cu and Zn were found in the muscles of red deer from the south-western region (namely, 2.99 and 25.78 mg kg^−1^) than those in animals from north-eastern Poland (namely, 2.61 and 23.39 mg kg^−1^ wet weight). In terms of human nutritional needs, the meat of red deer can be considered as a good source of Cu and Zn. Furthermore, Rb, Cs, Sr and Ba concentrations did not differ statistically between regions. Their mean concentrations were 4.50, 0.09, 0.16 and 0.31 mg kg^−1^ wet weight, respectively. Although high Cs, Sr and Ba concentrations were found, the meat of red deer does not pose a risk for adult consumers. Only high Ba content may potentially result in negative health effects for children.

## Introduction

Studies on the elemental composition of ruminant tissues are performed mainly on farm animals. There are relatively few data in the literature pertaining to wild ruminants, including red deer whose meat is valuable and a desired component of the human diet (Jarzyńska and Falandysz 2011a). The muscle meat of these animals is considered to be particularly culinary attractive; however, offal is also consumed. Food produced from the edible parts of game animals is common and can be found in market chains in both processed and raw form. The increasing popularity of game, commonly perceived as healthy meat produced without harmful additives, has expanded its range of consumers, which is not limited to hunters and their relatives. The number of red deer in Poland is increasing over time from 117,000 in year 2000 to 180,000 in 2010. Of the 164,000 animals registered in 2008, 46,000 were shot in hunting season 2008/2009, and the meat has been directed on the national market and for export (Central Statistical Office 2011). Therefore, it is important to analyse the concentrations of selected metals in the edible tissues of these game animals, which would allow for assessing its impact on consumers’ health.

The red deer—a free-living animal—is closely associated with the natural environment. For this reason, the analysis of pollutants in its tissues can help to enable the assessment of animal exposition to selected toxic factors, which might affect the quality of the acquired meat. These factors include among others trace elements that may exert a toxic effect or may be beneficial. An important issue is the pollution of skeletal muscles of game animals by lead from bullets used in shooting because lead bullets, particularly shell burst type, are a secondary source of tissue contamination (Jarzyńska and Falandysz 2011a). Lead is a metal monitored in animal products, which is reflected by appropriate legal regulations (European Commission 2006). Due to its long half-life in tissues, the toxicity of this metal is of most concern when considering the potential risk to consumers’ health. Falandysz and Caboń (1990) were the first who noticed a high lead content in the meat of game animals and associated a health risk for consumers in Poland. In the case of cadmium, which is a pollutant commonly found in the environment, muscle tissue is not a good indicator of its presence in the body because it accumulates mainly in the kidneys and in a lesser extent in the liver (Jarzyńska and Falandysz 2011a). Although offal is treated as an edible part of the animal’s body, in practice in many cases, it is eliminated by veterinary service. The main reasons are its susceptibility to spoilage and the presence of different parasites. Therefore, the liver and kidneys of game animals have a marginal share in the consumption of game meats in comparison to muscle meats.

An important issue is the zinc and copper content in the meat of game animals because both metals are cofactors of many enzymes that are responsible for proper metabolism, and their deficiency as well as excess can have negative health effects (Frank et al. 2000; Prasad 2002; Kośla et al. 2004; Brewer 2005; Rink and Haase 2007; Zatta and Frank 2007). In contrast, there is a group of elements such as rubidium, cesium, strontium and barium with unclear metabolic functions. They are quite common in the environment and are able to replace important intracellular and extracellular cations in metabolic reactions. The possibility that they are essential has not yet been well documented (Kabata-Pendias and Pendias 1999; Kośla et al. 2002; Milman et al. 2006; Jarzyńska and Falandysz 2011a). In recent years, there has been increased interest in them, especially strontium. Experiments in animals have shown that strontium salts may have a potential benefit for the treatment of osteoporosis (Marie 2005).

The aim of this study was to determine the content of seven metals (Pb, Cu, Zn, Rb, Cs, Sr and Ba) in the skeletal muscles of red deer from north-eastern and south-western Poland, and then to relate these results to human nutritional demands and to the potential effects of these metals on the health status of consumers.

## Material and methods

**Fig. 1:**
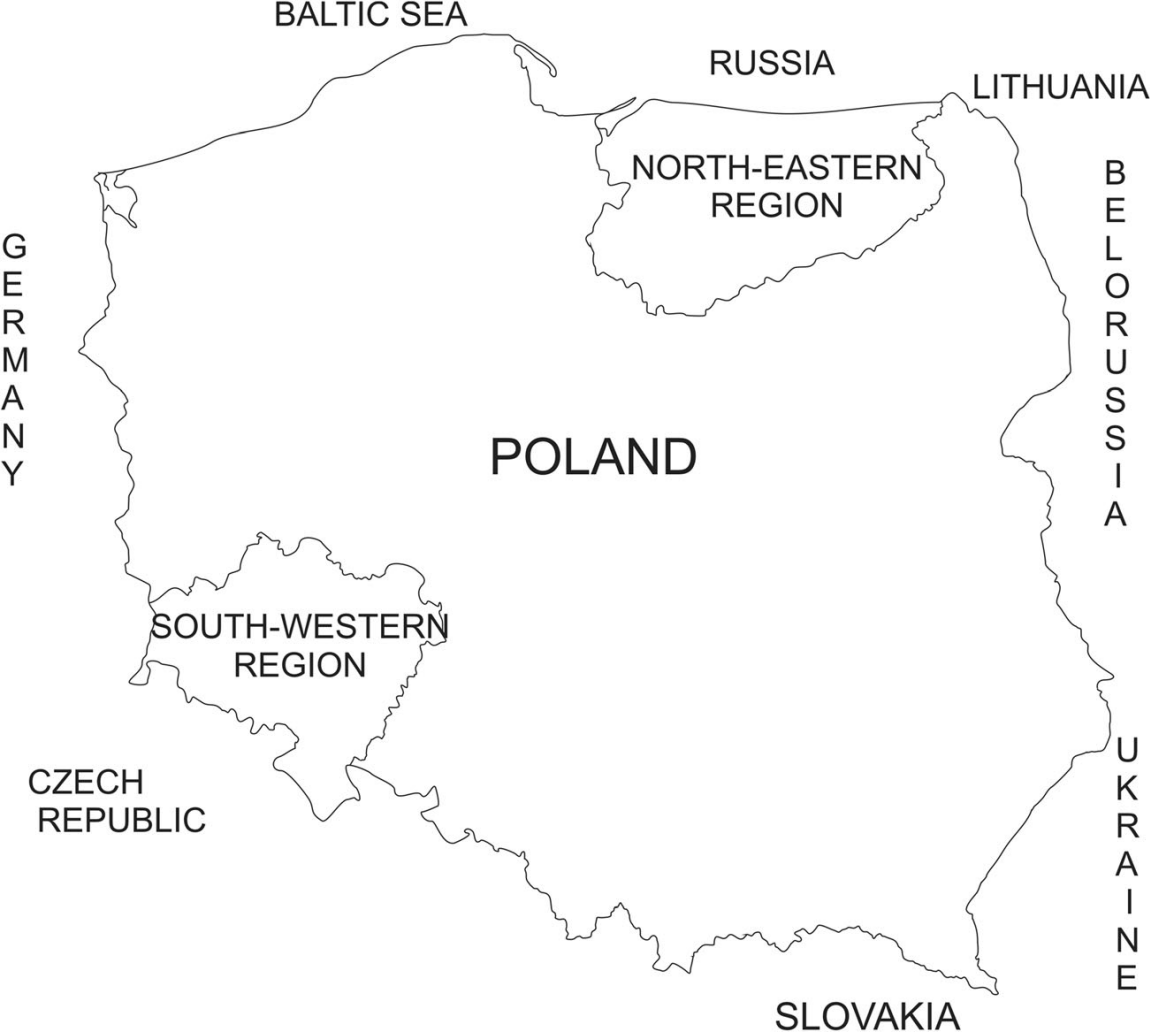
Sampling sites according to the geographical location

The study material consisted of samples of muscles collected from 50 hunted female red deer (*Cervus elaphus*) from south-western (23 individuals) and north-eastern (27 individuals) Poland (Fig. 1). The south-western region represented Lower Silesian voivodeship neighbouring Germany and the Czech Republic. In the 1990s, this region was under the influence of industrial emissions from the three neighboring countries. Currently, this region has achieved considerable improvement in its environment state. The north-eastern Poland is represented by the Warmia and Mazury regions bordering with Russia and Lithuania. North-eastern Poland is a region of low industrialization with many protected areas, which serves as a refuge for numerous species of the cervine family. Both regions are covered with a similar percentage of forests. For example in the province of Lower Silesia, the forest cover is 29.6 %, while in Warmia and Mazury, it is 30.8 % (The State Forests National Forests Holding 2011).

Study material was collected in the years 2008 and 2009 in game processing factories. Animals were shot during the hunting season between 1 October 2008 and 15 January 2009, in compliance with the hunting limit set. The animals were in the same age range of 4 to 6 years. Because of the character of the study that required the collection of post mortem tissues, no consent of ethical commission was necessary according to Polish law.

Samples of the muscles of about 10 g were collected during examination by veterinary service. The study material comprised sections of both superficial and deep part of the *m. masseter*. It is a large unit allowing an easy access and collecting of an adequately representative sample. Its weight is significantly correlated with body weight, amounting in all ruminants to approximately 0.2 %. This muscle is characterized by high contractile activity and belongs to the largest muscular units involved in mastication (Axmacher and Hofmann 2009). Because of its proximity to the large blood vessels and lymph nodes of the head, there is a probability that certain metals may pass to this muscle directly from the oral cavity and then into the general circulation without the effect of the liver on their absorption and distribution.

Until the time of chemical analyses, samples were frozen and stored in polyethylene bags at −20 °C. Before the beginning of chemical analyses, they were homogenized, and then, 0.5 g of the sample was placed in high-pressure Teflon containers.

Mineralization was performed in a Milestone microwave system. The content of elements in muscle samples was determined with the inductively coupled plasma–mass spectrometric method (ICP-MS, ELAN DRC II, Perkin Elmer, USA). The method was validated with certified reference material CRM-BCR 184 R (Community Bureau of Reference, BCR in Brussels, Belgium). Discrepancies between the certified values and concentrations quantified were below 10 %. All analyses were performed in triplicate, and the results are presented in Table 1 as the means of measurements expressed in mg kg^−1^ wet weight.

**Table 1:** The mean concentration of lead and other metals in muscles of the red deer tested according to the geographical location (mg kg^−1^ wet weight)

Region	N		Mean ± SD	Median	Min	Max
South-western (1)	Pb	23	0.12 ± 0.10	0.09	0.04	0.47
	Cu	23	2.99* ± 0.48	3.08	2.04	3.67
	Zn	23	25.78* ± 2.83	26.00	18.00	31.00
	Rb	23	5.03 ± 1.37	5.11	2.59	7.98
	Cs	23	0.09 ± 0.08	0.07	0.04	0.39
	Sr	23	0.18 ± 0.08	0.20	0.09	0.30
	Ba	23	0.33 ± 0.23	0.25	0.11	1.01
North-eastern (2)	Pb	27	0.10 ± 0.09	0.07	0.04	0.48
	Cu	27	2.61* ± 0.54	2.75	1.31	3.53
	Zn	27	23.33* ± 3.91	23.00	16.0	32.0
	Rb	27	4.05 ± 2.09	3.04	1.73	9.30
	Cs	27	0.08 ± 0.08	0.04	0.04	0.37
	Sr	27	0.15 ± 0.06	0.10	0.09	0.30
	Ba	27	0.29 ± 0.19	0.25	0.11	1.05
Total	Pb	50	0.11 ± 0.10	0.08	0.04	0.48
	Cu	50	2.79 ± 0.55	2.83	1.31	3.67
	Zn	50	24.50 ± 3.64	25.00	16.00	32.00
	Rb	50	4.50 ± 1.85	4.45	1.73	9.30
	Cs	50	0.09 ± 0.08	0.05	0.04	0.39
	Sr	50	0.16 ± 0.07	0.20	0.09	0.03
	Ba	50	0.31 ± 0.21	0.25	0.11	1.05

*p ≤ 0.05, statistically significant

The obtained results were subject to statistical analysis using Statistica 10.0™ software (StatSoft, Inc., Kraków, Poland). Initially, the data was investigated to determine their distribution using the Kolmogorov–Smirnov test. The concentrations of elements had normal distribution, and therefore, the differences were tested with parametric methods. Statistical analysis of the obtained results was conducted based upon the one-way analysis of variance, ANOVA module. The origin of animals was used as a grouping variable. For statistical comparisons between the particular groups, the Tukey test was used. The differences were considered to be significant at *p* ≤ 0.05 or highly significant at *p* ≤ 0.01. Correlations between the concentrations of particular elements were determined with the Pearson correlation coefficients. Statistical significance of the correlation coefficients was tested at the level *p* ≤ 0.05 and *p* ≤ 0.01.

## Results and discussion

Descriptive statistics of determined metals (Pb, Cu, Zn, Rb, Cs, Sr and Ba) are shown in Table 1. The concentrations of Pb, Rb, Cs, Sr and Ba did not differ statistically between muscles obtained from both regions. From the essential trace elements analysed, only concentrations of Cu and Zn were significantly different between groups (Table 1). Data on their mutual relationships are presented in Table 2. When analysing the correlations between lead and essential metals, it was observed that in the south-western region, it was positively correlated with Cs and Ba. In the north-eastern region, Pb was significantly correlated with Sr, Ba and Cu and highly significant with Cs. Most of the significant associations between essential metals were found in the north-eastern region, these correlations being highly significant between Ba-Sr and Cs-Rb. Rb was also correlated with Zn although the significance of this association was quite low. In the north-eastern region, there was positive correlation between Cu and Zn.

**Table 2:** Correlation between the concentration of chosen metals in the muscles of red deer according to the geographical location

Region		Sr	Cs	Ba	Pb	Cu	Zn
South-western (1)	Cs	0,0673					
	Ba	0,0156	0,4059				
	Pb	−0,1405	0,4533*	0,4531*			
	Cu	0,2198	0,0616	0,2430	0,0765		
	Zn	−0,1315	−0,0869	−0,1740	−0,0138	0,0277	
	Rb	0,2311	−0,0663	−0,0849	−0,1491	0,4210*	0,0327
Region		Sr	Cs	Ba	Pb	Cu	Zn
North-eastern (2)	Cs	−0,1383					
	Ba	0,5549**	−0,1371				
	Pb	0,4675*	0,5139**	0,5301*			
	Cu	0,0871	0,2241	0,2010	0,4248*		
	Zn	−0,0975	0,1407	0,1200	0,1304	0,6579*	
	Rb	−0,1788	0,6091**	−0,3591	0,1375	0,2761	0,3884*

Correlation coefficient significant at: *p ≤ 0.05; **p ≤ 0.01

### Pb, Cu and Zn

The lead content in analysed muscle tissue was almost twice that found previously by Jarzyńska and Falandysz (2011a). In red deer from Warmia and Mazury, they noted 0.05 mg Pb kg^−1^ fresh weight of analysed muscles. In other studies from the same region, Falandysz et al. (2005) recorded mean lead concentrations of 0.22 mg kg^−1^ fresh weight of muscles—the value two times higher than our results. There are no data on tolerable concentrations of lead in the tissues of game animals. Present legal regulations pertain only to the lead content in the meat of farm animals. The lead content in the meat of cattle, sheep, pigs and poultry (excluding giblets) should not exceed 0.1 mg kg^−1^ fresh weight (European Commission 2006). The mean content of lead found in this study equalled that permitted for farm animals while the lead content in four animals greatly exceeded the standard. The highest value observed in the south-western region was 0.47 mg kg^−1^ fresh weight, while in the north-eastern region, it amounted to 0.48 mg kg^−1^ fresh weight. Due to the type of analysed muscles, these results should be considered high because the sections of *m. masseter* analysed and the obtained heads did not show gunshot wounds. Usually, the use of lead bullets is responsible for secondary lead contamination; therefore, tissues surrounding wounds should be carefully removed together with the margin of unaffected tissue (Jarzyńska and Falandysz 2011a). Therefore, explanation of this relatively high lead concentration in the analysed muscles, which were not secondarily contaminated, may be due to the presence of lead in food and its passage through the mucous membrane of the oral cavity directly to the adjacent tissues, e.g. via lymphatic system. The results of these studies show that (apart from meadow lichen, tree branches and cereal) various species of fungi present in the environment and eaten by the red deer may be bio-concentrators of metals (Jarzyńska and Falandysz 2011b; Falandysz et al. 2007, 2011). According to WHO (1993), the safe level of lead intake for the health of an adult person daily varies from 0.21 to 0.25 mg. The share of particular lead sources in the total daily intake significantly differs among countries. In Poland, 90 % of lead intake comes from vegetables, cereal products, meat and meat products (Marzec and Schlegel-Zawadzka 2004). Data from EFSA (2010) estimates that the mean lower and upper level of daily lead exposure in Poland is 0.67 and 1.14 μg kg^−1^ body mass, respectively. Furthermore, for these doses, 7.91 % originates in meat and meat products. Studies on the effect of particular type of diet on lead consumption showed that the diet from game animals results in a 2.5 times higher exposure compared with a typical diet. Results obtained in this study show that the meat of the analysed animals does not pose a risk of lead intoxication. For example, consumptions of 100 g result in the intake of 0.01 mg Pb, which is less than 5 % of daily lead dose allowed by WHO (1993) and an amount smaller than data from EFSA (2010) estimates.

Mean concentrations of copper and zinc (Table 1) were 24.46 and 0.107 mg kg^−1^ fresh weight, respectively. Higher concentrations of Cu and Zn were found in the muscles of individuals from south-western Poland compared to the north-eastern region. Differences between groups of animals were statistically significant (*p* ≤ 0.05) with the differences probably resulting from biogeochemical conditions and from a smaller content of copper in the soils of north-eastern Poland. An essential part of the natural red deer food in rich habitats, depending on the season, is a deciduous browse. In poorer habitats in the spring and summer, red deer eat large quantities of grasses and herbs, while the food basis in autumn is the browse derived from coniferous species. In addition to forest and meadow plants, crop plants such as beets, potatoes and grains are also a significant part of the red deer diet (Kamler and Homolka 2005). Fields commercially used by farmers are fertilized by manure obtained from pig farms where the animals generally receive large doses of Zn and Cu in their feed (Jacela et al. 2010). This raises the question of the potential impact on the concentrations of these metals in plants growing in these fields and their transfer to the tissues of game animals. Studies of arable soils in the north-eastern region performed in years 2005–2007 showed no contamination with copper and zinc. In 4 among 20 control points located in the south-western region, high concentrations of both metals were found. In 2 points, the soil was contaminated to the 4th degree (Terelak et al. 2008). According to the data published by the Central Statistical Office (2011), the number of pigs in the years 2008–2009 differed significantly between the two regions. In the south-western Poland, it amounted to 302,000, while in the north-eastern region 670,000. Therefore, the higher Zn and Cu concentrations in muscles of the red deer from south-western Poland are more the result of industrial pollution than the agricultural production.

Our results fall within the range of previous values recorded for red deer in Poland and other regions of Europe (Gasparik et al. 2004; Bilandzic et al. 2009). Jarzyńska and Falandysz (2011a) in their analyses of elemental composition of skeletal muscles of red deer from Warmia and Mazury (NE Poland) found slightly higher mean concentrations that amounted to 3.3 and 45 mg kg^−1^ fresh weight of Cu and Zn, respectively. In other studies of red deer from the region, copper concentrations varied from 1.9 to 6.4 mg kg^−1^ and zinc concentrations from 19 to 64 mg kg^−1^ fresh weight (Falandysz et al. 2005). Copper is the essential microelement as cofactor for enzymes determining the proper course of many metabolic processes and including the peroxide dismutase, ceruloplasmine, cytochrome oxidase, lysine oxidase, tyrosinase and dopamine hydroxylase enzymes. The copper also plays a key role in iron metabolism (Frank et al. 2000; Brewer 2005; Zatta and Frank 2007; Skibniewska et al. 2010, 2012a).

Zinc is a cofactor of more than 300 enzymes like lactate dehydrogenase, alkaline phosphatase and carbonate anhydrase. The element takes place in protein and carbohydrate metabolism, in healing injured tissues, reproduction, respiration, seeing and in proper functioning of the kidneys (Prasad 2002; Kośla et al. 2004; Skibniewska et al. 2013a). It is a component of nuclear receptors of steroid and thyroid hormones (Drake and Sky-Peck 1989). An extremely important role of zinc is its participation in the immune response, and zinc deficits manifest themselves in immunodeficiency (Rink and Haase 2007).

Safe and appropriate dose of ingested copper for human organism is about 5 mg day^−1^, out of which 2 mg is absorbed directly from alimentary tract and is transported to blood, which distributes copper ions in tissues (Zatta and Frank 2007). The daily demand for zinc in an adult person is estimated at 10 mg (Kabata-Pendias and Pendias 1999). Consuming a portion of 100 g meat obtained from a studied individuals is equivalent to the ingestion of 0.27 mg Cu and 2.44 mg Zn. These doses cover 13 % of daily demand for copper absorbed from alimentary tract and nearly one fourth of the daily demand of an adult person for zinc. Therefore, the meat of red deer is a good source of both metals.

### Rb and Cs

Rubidium is an alkaline metal of exceptional chemical activity. Together with lithium, sodium, potassium and cesium, it belongs to the first group of periodical table of elements. Due to being widespread, rubidium may substitute potassium in metabolic reactions, which may disturb their proper course. Even though the importance of rubidium for animals is not clearly understood, studies performed so far have indicated that Rb may be an essential element but the margin between its necessary and toxic level is very narrow (Angelow 1994; Kośla et al. 2002; Milman et al. 2006; Skibniewska et al. 2012b, 2013b). The mean content of rubidium in muscles of analysed red deer was lower than that reported by Jarzyńska and Falandysz (2011a). According to their data, the mean content of rubidium in red deer from north-eastern Poland was 19 mg kg^−1^ dry weight or 5.7 mg kg^−1^ fresh weight.

The mean cesium content found in this study was almost two times higher than the 0.045 mg kg^−1^ fresh weight obtained by Jarzyńska and Falandysz (2011a). Nevertheless, the level should be considered safe for consumers. There are few data in the literature on the effect of stable cesium on human health. Cesium, like rubidium, is antagonistic to potassium, and the metal is mainly accumulated in soft tissues (Kabata-Pendias and Pendias 1999). Its higher concentrations may be found in skeletal muscles, and as potassium, it is mainly localized in intracellular fluids (ATSDR 2001a). There is a description in the literature of a volunteer, who took up 68 mg cesium kg^−1^ body mass daily for 36 days, and the observed symptoms pertained mainly to the alimentary tract and included the following: the loss of appetite, nausea and diarrhoea, which retreated shortly after the intake of high potassium doses (Neulieb 1984).

### Sr and Ba

Strontium is considered an essential element participating in the transformation of calcium and phosphorus. A beneficial effect of low doses of stable strontium in the treatment of osteoporosis was described more than 60 years ago. Since that time, early experiments on rodents that received moderate amounts of strontium chloride started. Despite numerous studies, the detailed cellular mechanisms underlying the beneficial effect of low strontium doses on bone formation are still not completely known (Dahl et al. 2001). Strontium is ingested the same way as calcium, and studies on humans showed that only 20 % of ingested strontium is absorbed from the alimentary tract (ATSDR 2001b). Experiments with volunteers have indicated that its absorption from the gut takes place by an active transport mechanisms involving a calcium-binding protein (Leeuwenkamp et al. 1990). Thus, it has been suggested that excessive doses of strontium could disturb calcium metabolism. At higher doses and inappropriate levels of Ca and P in the diet, bone formation may be disturbed, which manifests itself by strontium rickets in children (Özgür et al. 1996). Experiments on rats have shown that mechanisms decreasing uptake of Sr develop during maturation. It is of interest to note, therefore, that the high efficiency of strontium absorption by the gastrointestinal tract in young mammals may be due to a deficiency of absorptive discrimination of Sr in favour of Ca (Sugihira and Suzuki 1991). At levels normally encountered in the natural environment, strontium appears to have low toxicity. No unfavourable symptoms of its activity have been observed at daily doses smaller than 100 mg kg^−1^ when accompanied by appropriate levels of calcium, phosphorus and vitamin D3 (ATSDR 2001b). The daily doses of strontium administered in humans during treatment of osteoporosis, which were considered low, amounted to 316–634 mg kg^−1^. A significant increase in the lumbar vertebrae density of osteoporotic women was observed after 1 year of treatment with 517 mg of stable strontium daily administered in the form of strontium ranelate (Dahl et al. 2001).

The mean strontium content of 0.164 mg kg^−1^ fresh weight found in this study was definitely higher than the 0.039 mg kg^−1^ value obtained by Jarzyńska and Falandysz (2011a). A meal consisting of a 100 g meat obtained from a studied individuals will provide a small dose in comparison to amounts of strontium administered during treatment of osteoporosis. Nevertheless, it can be treated as an additional source of this element, which appears to be beneficial for adults, particularly for woman in postmenopausal period.

Barium in mammals accumulates mainly in the bone tissue, and according to Kabata-Pendias and Pendias (1999), its daily intake by humans is about 0.5 mg. According to Canadian studies, daily dose of barium varies depending on age. In children up to 4 years old, it is from 20.7 to 25.2 μg kg^−1^ body mass while in persons above 65 years of age, it is only 7.5 μg kg^−1^ (Health 2005). These differences are closely associated with a decreasing ability to absorb soluble forms of Ba^2+^ administered orally, which is differentiated in humans and animals. The absorption ranges from 3 to 60 % of the given dose (Dallas and Williams 2001). Studies by the International Commission for Radiological Protection showed evidence that the effectiveness of barium absorption in humans decreases with age. Only 20 % of a given barium is absorbed in adults and 30 % in children from 1 to 15 years old while children younger than 1 year of age absorb 60 % of the dose (ICRP 1993). According to data published by IRIS (2006), the maximum tolerated daily dose (TDI) of barium is 0.2 mg kg^−1^ body mass, which is much more than the amounts given by Kabata-Pendias and Pendias (1999). The mean concentration found in our study (0.309 mg kg^−1^ fresh weight) should be considered high as it was 6.4 times higher than that obtained by Jarzyńska and Falandysz (2011a), which was 0.16 mg kg^−1^ dry weight or 0.048 mg kg^−1^ fresh weight. High concentrations of barium in the analysed muscles is not harmful for adults but may have negative health effects in children.

## Conclusions

Concentrations of most metals (with the exception of copper and zinc) did not differ significantly in individuals from the two study regions. The differences between Zn and Cu concentrations resulted from biogeochemical conditions and from a smaller content of both metals in the soils of north-eastern Poland. The meat of the red deer may be dealt with as an appropriate source of Cu and Zn in the human diet. Furthermore, the observed levels of lead do not pose a threat to consumers’ health. In spite of taste values and a common conviction of a high quality of game meats, one should give it to children with caution because of high barium contents that when combined with the effective absorption of the element by the young may potentially result in negative health effects.
